# Supplementary material for: Necrotic Cell Death and Inflammasome NLRP3 Activity in Mycobacterium bovis-Infected Bovine Macrophages
Source: Cells. 2023 Aug 17;12(16):2079. doi: 10.3390/cells12162079 (PMC10453650; doi:10.3390/cells12162079)
Supplement: Supplementary file 1 [file cells-12-02079-s001.zip › cells-2503360-supplementary.pdf]

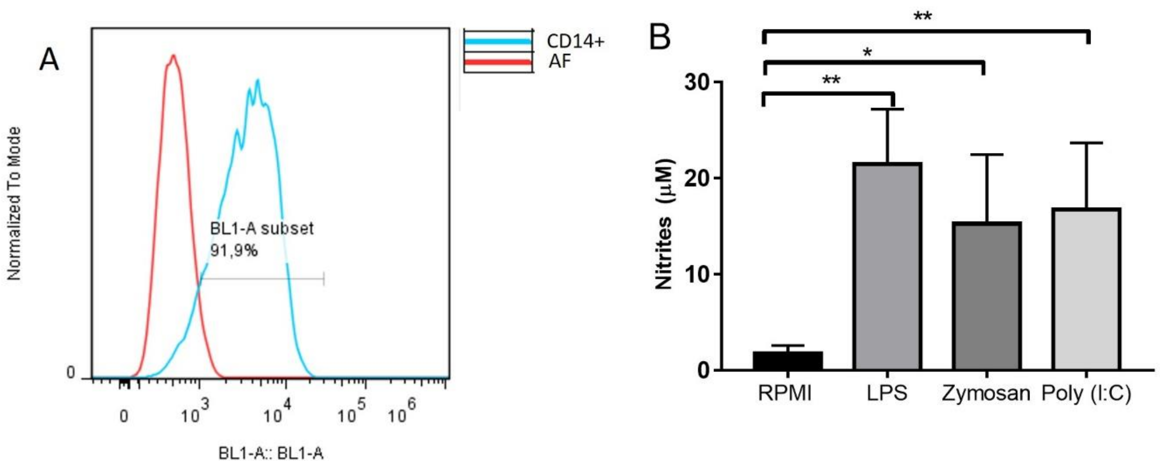

Figure S1. Characterization of macrophages derived from bovine peripheral blood. **(A)** Detection of CD14-positive cells by flow cytometry. **(B)** Nitrite production in macrophages stimulated with various ligands (300 ng/ml LPS, 30 µg/ml Poly I:C, and 30 µg/ml Zymosan). Results are shown as the mean ± S. D. of three independent experiments with three internal replicas each. One-way ANOVA showed significant differences between negative control (RPMI) versus treated cells. * *p* value ≤ 0.05, ** *p* value ≤ 0.01.

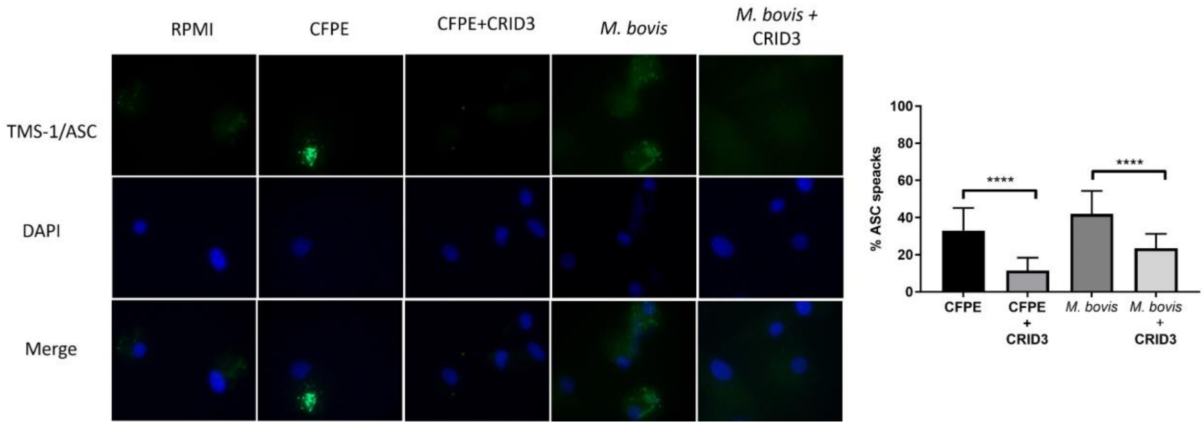

Figure S2. ASC specks were inhibited by CRID3. ASC specks in macrophages stimulated with CRID3 + 100 µg/ml culture filtrate extract (CFPE) in $1\times10_{6}$ cells or *M. bovis* MOI 10:1. Fluorescence microscopy with a 40× objective was used to quantify ASC specks. ASC specks are shown in green, and the cell nucleus is shown in blue.

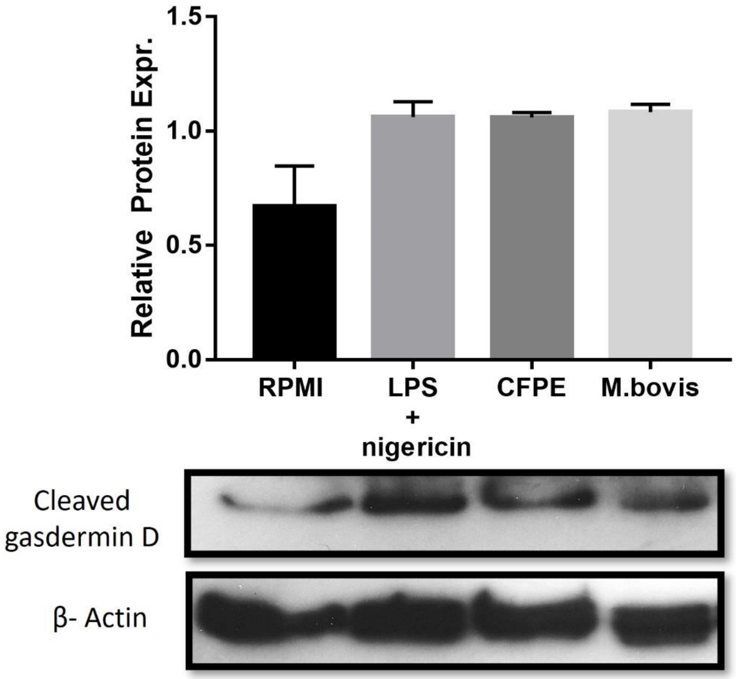

Figure S3. Cleaved gasdermin D induced by *M bovis* and CFPE in macrophages. Bovine macrophages were infected with *M. bovis* AN5 MOI 10:1 or treated with CFPE (100 µg/ml) for 24 h. LPS (300 µg/ml) + nigericin (50 µM) was used as a positive control. The concentration of cleaved gasdermin D was monitored by immunoblot. Protein content load was monitored with anti-β actin. The results represent two independent experiments and are expressed as the ratio of cleaved gasdermin D/β actin.

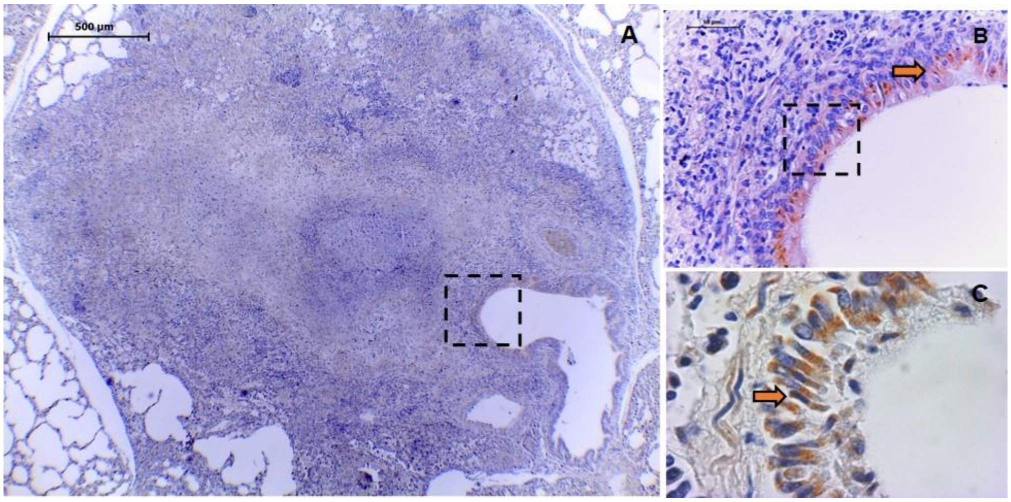

Figure S4. Immunohistochemistry of cleaved gasdermin D and *Mycobacterium bovis*. **(A)** Stage III granulomas in the lung, showing positive areas to gasdermin D. **(B)** Amplification of area; the marks show positive epithelial cells, and the arrow shows positive staining. **(C)** respiratory

epithelial cells with abundant anti-mycobacterium staining; the arrow shows positive staining. Results are shown as the mean ± S. D. of three independent experiments with three internal replicas each.

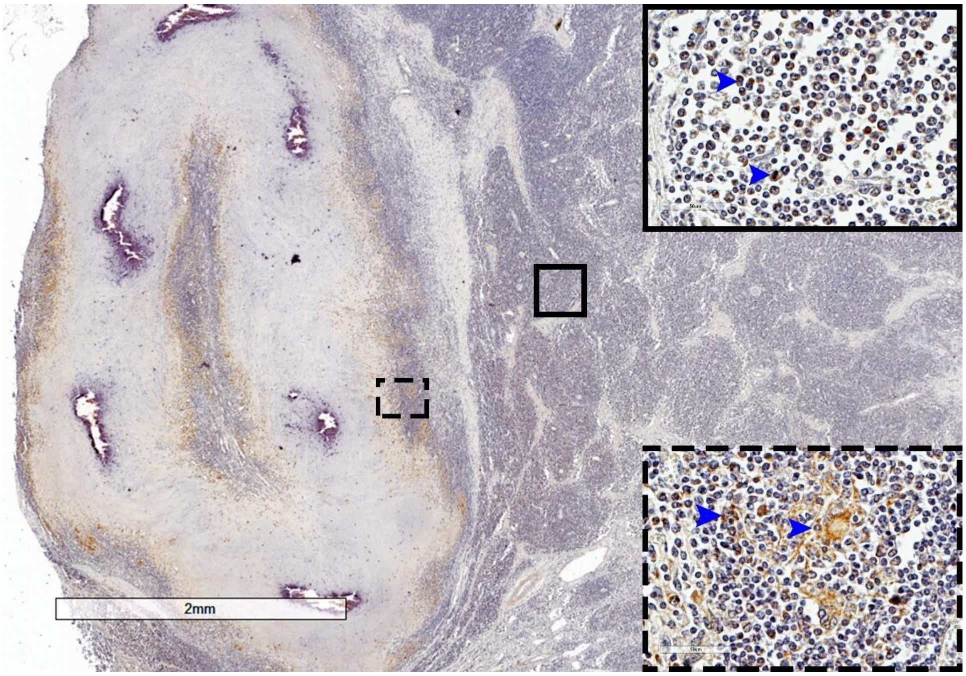

Figure S5. Immunohistochemistry of lymph node with granuloma from a cow naturally infected with *M. bovis*. Immunolabeling of mycobacteria inside and outside of the lesion. Microphotograph of anti-mycobacterium IHC, with stage IV adult bovine granuloma with faint positive brown areas around the necrosis, close-up in box with the dotted line encloses cells with macrophagic morphology and a giant cell with positive staining, and the continuous line delimits cells with positive staining outside the lesion.

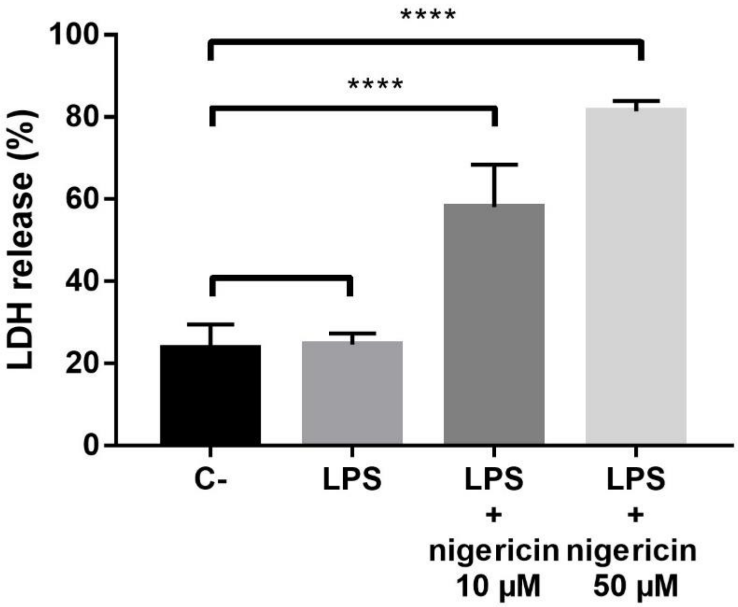

Figure S6. Induction of cell death with LPS + nigericin. Bovine macrophages were stimulated with 300 μg/ml LPS + 10 μM and 50 μM nigericin for 24 h. Results are shown as the mean ± S. D. of three independent experiments with three internal replicas each. The groups were tested for statistical differences with a one-way ANOVA. * *p* value ≤ 0.05, **** *p* value ≤ 0.0001.
